# Efficacy of Bravecto^®^ Plus spot-on solution for cats (280 mg/ml fluralaner and 14 mg/ml moxidectin) for the prevention of aelurostrongylosis in experimentally infected cats

**DOI:** 10.1186/s13071-021-04610-y

**Published:** 2021-02-16

**Authors:** Katharina Raue, Nadja Rohdich, Daniela Hauck, Eva Zschiesche, Simone Morelli, Donato Traversa, Angela Di Cesare, Rainer K. A. Roepke, Christina Strube

**Affiliations:** 1 Institute for Parasitology, Centre for Infection Medicine, University of Veterinary Medicine Hannover, Hanover, Germany; 2 MSD Animal Health Innovation GmbH, Schwabenheim, Germany; 3 Faculty of Veterinary Medicine, University of Teramo, Teramo, Italy

**Keywords:** *Aelurostrongylus abstrusus*, Feline lungworms, Macrocyclic lactones, Moxidectin, Treatment, Prevention, Control

## Abstract

**Background:**

The feline lungworm *Aelurostrongylus abstrusus* affects the lower respiratory tract in cats worldwide. As infections may lead to chronic respiratory changes or even death, preventive treatment in cats with outdoor access is warranted.

**Methods:**

The preventive efficacy of a spot-on solution (Bravecto® Plus spot-on solution for cats, MSD) against cat aelurostrongylosis was evaluated using three different preventive treatment regimes in a negative controlled, randomized and partially blinded laboratory efficacy study with 31 purposed-bred cats. The minimum recommended dose of 2.0 mg moxidectin + 40 mg fluralaner/kg bodyweight was applied once 12 (Group [G]1), 8 (G2) or 4 (G3) weeks before experimental infection with 300 third-stage larvae (L3) of *A.* *abstrusus*. Another group served as untreated control (G4). Individual faecal samples were analysed as of day 30 post infection (pi) to monitor larvae excretion. Necropsy was performed at days 47–50 pi. The lungs were examined macroscopically for pathological findings and (pre-)adult worms were counted to assess preventive efficacy.

**Results:**

Beginning at day 32–40 pi, all cats of the control group were constantly shedding larvae of *A. abstrusus*, whereas only one animal of G1 excreted larvae at several consecutive days. In addition, two cats of G1 and G3 and three of G2 were positive on a single occasion. The geometric mean (GM) of the maximum number of excreted larvae was 7574.29 in the control group compared to 1.10 (G1), 1.19 (G2) and 0.53 (G3), resulting in a GM reduction of > 99.9% in all treatment groups. All lungs of the control animals showed severe or very severe alterations at necropsy, while in 94.44% of the treated cats lung pathology was rated as absent or mild. The GM number of (pre-)adult *A. abstrusus* retrieved from the lungs was 26.57 in the control group, 0.09 in G1 and 0.00 in G2 and G3. Thus, GM worm count reduction was 99.66% in G1 and 100% in G2 and G3.

**Conclusions:**

A single application of Bravecto® Plus spot-on solution at a dose of 2.0 mg moxidectin + 40 mg fluralaner/kg bodyweight reliably prevents cat aelurostrongylosis for at least 12 weeks.
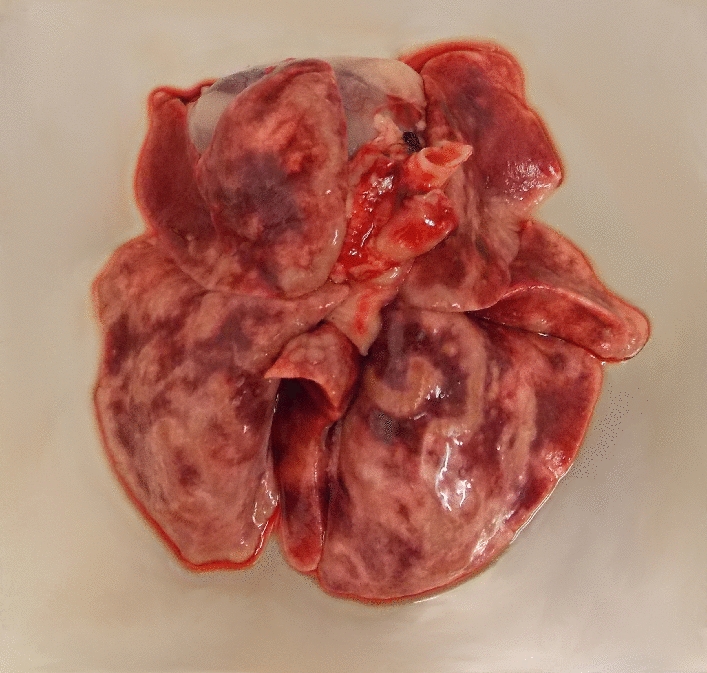

## Background

The cat lungworm *Aelurostrongylus abstrusus* Railliet, 1898 (Nematoda, Metastrongyloidea) causes mild to severe respiratory disease in cats with clinical signs such as coughing, sneezing, nasal discharge, panting, respiratory sounds and dyspnoea [1–3] and occasionally leads to death [4]. However, patent infections may be also detected during routine faecal examination in sub-clinically infected cats [2, 5, 6]. The heteroxenous life-cycle relies on gastropods as intermediate hosts, and different prey species play a role in transmission as paratenic hosts [7, 8]. Most reports on *A. abstrusus* prevalence in cats originate from Europe, ranging between 0.1 and 10.7% in northern and central Europe [9–16] and between 8.0 and 27.3% in eastern and southern Europe [5, 17–20], but studies were also conducted in Asia (e.g. Qatar 7.5% [21]), the Americas (e.g. Argentina 2.6% [22], Midwest USA 1.2%, Northeast USA 2.7% [23]) and Australia (e.g. Christmas Island 25.0% [24]). A supposed risk factor for *A.* *abstrusus* infection is young age [5, 13, 19, 23, 25], potentially due to an immature immune system [23, 26]. Other factors that have been linked to a higher prevalence are neutering status [5, 13], FIV infection [25] and outdoor access [5, 10, 13].

Due to non-specific signs in combination with methodological limitations, the clinical diagnosis of aelurostrongylosis is challenging. Detection of first-stage larvae (L1) by copromicroscopy, e.g. via the Baermann migration method, may be hampered by irregular larval shedding [1] and presumably low survival rates of excreted larvae in faeces mixed with cat litter [27]. In addition, the collection of faecal samples may be difficult, especially in cats with outdoor access, which are particularly at risk of acquiring *A. abstrusus* [5, 10, 13]. An ELISA for the detection of serum antibodies against *A. abstrusus* using recombinantly expressed bovine lungworm major sperm protein (MSP) as diagnostic antigen [28] has been recently developed [26]. However, this serological test is being used only in experimental and epizootiological studies so far [13, 18, 20, 25].

Currently, products licensed in Europe for the treatment of cat aelurostrongylosis contain fenbendazole (only in the UK), emodepside, moxidectin or eprinomectin. In addition, some data suggest the potential efficacy, in terms of resolution of clinical signs and termination of larval excretion, of selamectin and milbemycine oxime in single clinical cases reviewed in Refs. [29] and [30]. Effective parasite control should aim to preventing a patent infection and manifest disease. This is particularly true for *A. abstrusus* as lung damage occurs already during prepatency [31]. In this regard, an experimental study provided encouraging results, as eprinomectin was able to prevent clinical aelurostrongylosis and larval shedding when applied as early as 4 days post infection [32] and led to the license of a spot-on formulation containing eprinomectin against fourth- (L4) and fifth- (L5) stage larvae of *A. abstrusus*. Likewise, a product containing moxidectin is licensed against third-stage larvae (L3) and L4 if applied at monthly intervals, since it has been demonstrated that two moxidectin treatments 4 weeks apart at different time points before and after experimental infection reliably eliminate larval stages of *A. abstrusus* and hence can prevent lung damage and a patent infection [33]. Indeed, the long-lasting property of moxidectin makes this molecule a promising candidate for the prevention of aelurostrongylosis even after a single treatment several weeks prior to infection. Thus, the aim of the present study was to evaluate the efficacy and safety of a combination of moxidectin and fluralaner in preventing clinical aelurostrongylosis and patent infections in cats experimentally challenged with *A. abstrusus* at different time points (i.e. 4, 8 or 12 weeks) after a single treatment.

## Methods

### Study design

To evaluate the preventive efficacy of a single dose of 2.0 mg moxidectin + 40 mg fluralaner/kg bodyweight (BW) as a spot-on solution against cat aelurostrongylosis, the substance (Bravecto® Plus spot-on solution for cats) was applied topically 4, 8 or 12 weeks prior to experimental inoculation with infective *A. abstrusus* L3 in a negative (placebo-)controlled, randomized and partially blinded laboratory study. The study was conducted in accordance with VICH guideline 9 “Good Clinical Practice” [34] and VICH guideline 7 “Efficacy of anthelmintics: General requirements” [35].

### Study animals and group allocation

Thirty-one purposed-bred European Shorthair cats (16 females and 15 males) aged between 21–29 weeks with a mean bodyweight of 2.9 kg (2.2–4.2 kg) were purchased from a commercial breeder and acclimatized to the study site (Institute for Parasitology, University of Veterinary Medicine Hannover, Germany) for 7 days (study days [SD] -7 to -1) before inclusion in the study. For study inclusion, animals had to meet the following criteria: clinically healthy status, bodyweight ≥ 1.2 kg on SD 0, at least two faecal samples collected during the acclimatization period negative for the presence of nematode eggs or larvae (tested by a combined sedimentation-flotation technique with zinc sulphate as flotation solution [specific gravity 1.3] and the Baermann method) as well as no treatment with drugs that could interfere with the establishment of the experimental infection (e.g. long-acting anthelmintics). To achieve a homogeneous distribution of sex and body weight in all study groups, all 31 cats were ranked, prior to SD -7, by descending bodyweight, stratified by sex and allocated to the study groups according to a pre-defined list (order of the 16 female cats: 4-3-2-1-4-2-1-4-3-2-2-4-2-1-4-3, order of the 15 male cats: 1-2-3-4-3-1-4-1-2-3-1-3-4-1-2). Detailed information on the individual study cats and composition of the study groups at SD 0 is given in Additional file 1: Table S1.

Animals were housed indoors in environmentally controlled rooms in pairs or groups of three cats in their respective study groups. At the day of treatment and the 2 following days, and the day of experimental infection and for collection of individual faecal samples, cats were separated by dividing the pen with acrylic glass but still allowing audio-visual and olfactory contact with their group mates. Pens were equipped with litter boxes and bowls for food and water and were environmentally enriched with shelfs, scratch poles, toys and cardboard boxes. The animals received a standard commercial dry diet at recommended rates, and water was provided *ad libitum*.

### Health monitoring

General health observations were performed at least once a day by qualified and trained animal caretakers. The animals were weighed and physically examined by a veterinarian on the day before the treatment, before experimental infection and additionally if any abnormality was reported by the animal caretakers. An in-depth clinical respiratory assessment was performed on all cats the day before the first treatment and before experimental infection, respectively, and following that twice weekly until necropsy. The respiratory frequency, intensity of inspiratory and expiratory sounds on a scale from 0 to 3 (0 = no sound; 1 = slight sound; 2 = moderate sound; 3 = severe sound), quality of the respiratory sound (physiological sound, deepened but physiological sound, stertor, stridor, rhonchus, wheeze, crackle) and abdominal involvement, panting and coughing or retching (yes or no) were recorded.

### Treatment

Animals of groups (G) 1, 2 and 3 were dosed once with Bravecto® Plus spot-on solution at the minimum recommended dose on SD 0, SD 28 and SD 56, respectively. Animals of the control group (G4) were dosed with a placebo (0.9% saline solution) at the same study days (SD 0, SD 28 and SD 56). To maintain blinding, cats in G1 were treated with the placebo on SD 28 and SD 56, cats in G2 on SD 0 and 56 and cats in G3 on SD 0 and 28. An overview of the study design is given in Table 1.

**Table 1:** Overview of the study design to evaluate the preventive efficacy of Bravecto® Plus spot-on solution at the minimum recommended dose (2.0 mg moxidectin/kg BW + 40 mg fluralaner/kg) compared to placebo treatment (0.9% saline solution)

Group	No. of cats	Treatment (study day)		Infection with 300 *Aelurostrongylus abstrusus* L3 (study day)	Necropsy (study day)
1	8	0	Bravecto® Plus spot-on solution	84	131–134
		28	Placebo		
		56	Placebo		
2	8	0	Placebo		
		28	Bravecto® Plus spot-on solution		
		56	Placebo		
3	7	0	Placebo		
		28	Placebo		
		56	Bravecto® Plus spot-on solution		
4	8	0	Placebo		
		28	Placebo		
		56	Placebo		

Ten minutes and 24 h ± 4 h after treatment (i.e. SD 1 ± 4 h, SD 29 ± 4 h and SD 57 ± 4 h) and at the day of necropsy (SD 131 to 134), the administration site was checked for abnormalities. Additionally, 2 h ± 15 min after treatment all cats were observed by a veterinarian for general health. On all other days post-treatment, the cats were observed at least once a day for general health by a qualified animal caretaker (cf. section on health monitoring above).

### Experimental *Aelurostrongylus abstrusus* infection

Snail (*Cornu aspersum*) breeding, their infection with *A. abstrusus* L1 and snail maintenance until L3 development were conducted at the University of Teramo, Italy, as previously described [36]. All *C. aspersum* specimens originated from an established farm for heliciculture in Central Italy. The absence of natural infections of the snails with other metastrongylid nematodes was assessed morphologically and molecularly as previously described by examining 10% of the snails before starting their breeding [30, 36, 37]. Each snail was infected with 500 L1 of *A. abstrusus* obtained from faecal samples of a naturally infected cat. After infection, snails were kept at 26 °C and fed *ad libitum* with vegetables for at least 2 months to allow development of *A. abstrusus* L3 [36].

Digestion of the snails to obtain cat-infective L3 was performed at the day of infection (SD 84) based on an established protocol [38]. Briefly, the muscular foot of the snails was minced and transferred in freshly prepared digesting solution (6 g pepsin [≥ 2000 FIP-U/g, Carl Roth GmbH]) and 7 ml HCl 37% in 1 l water. The tissue of 10–15 snails each was added to 100–250 ml digesting solution and stirred in a water bath at 41 °C for 30 min. Then, the preparation was sieved and centrifuged at room temperature (600 *g* for 5 min). The supernatant was discarded, the sediment re-suspended in tap water and the centrifugation step repeated. The sediment was pooled, thoroughly shaken and placed on a magnetic stirrer. While stirring constantly, the L3 present in five aliquots of 0.1 ml suspension each were counted to determine the mean number of larvae in the respective volume. Afterwards, the volume of the inoculum containing approximately 300 L3 was calculated. Until inoculation of the cats, the larvae suspension was kept at 20–25 °C.

Each cat was inoculated orally with approximately 300 L3 of *A. abstrusus* on SD 84. For inoculation, the animals were anaesthetised by intramuscular injection of 80 µg/kg medetomidine (Domitor®, Vetoquinol) and 7.5 mg/kg ketamine (Ketamin® 10%, bela-pharm) combined in one syringe. Additionally, 0.3 mg/kg metoclopramide (Metomotyl® 5 mg/ml, CP-Pharma) was injected intramuscularly to avoid regurgitation. After applying the inoculum via a syringe connected to a stomach tube directly into the stomach, the tube was flushed with tap water and then pulled out. The animals were observed at 10 (± 2) min and 1 h (± 10 min) post inoculation if vomiting occurred. One cat in G2 (ID 7273) vomited approximately 1 ml fluid within this time frame and was re-inoculated with the complete dose (300 L3). Another 22 animals (7 in G1, 4 in G2, 4 in G3 and 7 in G4) vomited between 1 h after inoculation and the next morning but were not re-inoculated.

### Faecal larvae counts after experimental *A. abstrusus* infection

Individual faecal samples were collected from each cat daily between SD 114 and SD 130, i.e. between 30 and 46 days post infection (dpi). Samples of 5 g faeces each were processed with the Baermann method the same day or, if collected at the weekend, stored at 3–9.5 ˚C and processed the following Monday. L1 were allowed to migrate overnight and larvae counts were performed the next morning.

### Parasitological necropsy

On SD 131 to 134, i.e. 47 to 50 dpi, the animals were euthanized by intravenous application of pentobarbital (Euthadorm® 500 mg/ml, CP-Pharma) and necropsy was performed. Similar ratios of the study groups were euthanized and necropsied each day. After verification of death, the thorax was opened in the sternal region and the lungs were removed and examined macroscopically for any pathological findings. If present, these were rated from 0 = absent, 1 = mild, 2 = moderate, 3 = severe to 4 = very severe.

Afterwards, the trachea and bronchi were opened lengthwise with scissors and examined under a binocular loupe with 25× magnification for visible nematodes. Subsequently, the lungs were cut into small pieces (maximum 0.5 × 0.5 cm) and parasites were collected using dressing forceps and transferred into vessels containing 0.9% saline solution. Recovered nematodes were examined under a stereomicroscope with 40–100× magnification to determine viability, developmental stage and, if possible, sex. Dead worms were also classified as viable if they did not show signs of degradation. Worm fragments were counted only if the head or tail was present. The total *A. abstrusus* worm count per animal was calculated by summing the numbers of intact (pre-)adult worms and worm fragments (either heads or tails, whichever number was higher).

### Statistical analysis and calculation of preventive efficacy

Statistical analysis was performed using the SAS^®^ software package (version 9.4; SAS Institute Inc., Cary, NC, USA).

Primary preventive efficacy criterion was the geometric mean (GM) worm count in each treatment group compared to the control group. Necropsy worm counts were used to evaluate the percentage preventive efficacy in each treatment group using the following formula according to the recommendations for controlled tests described in VICH GL7 [35]:

$${\text{Efficacy reduction }}\left[ \% \right] \, = \frac{{\overline{x}_{{\text{C}}} {-} \, \overline{x}_{{\text{T}}} }}{{\overline{x}_{{\text{C}}} }} \times 100$$

where x̅_C_ is the GM number of *A. abstrusus* worms in the control group (G4); x̅_T_ is the GM number of *A. abstrusus* worms in each treatment group (G1–G3).

To allow the calculation in case of zero counts, the GM was calculated as follows:

$$x_{g} = \left( {\prod\limits_{i = 1}^{n} {(x_{i} + 1)} } \right)^{\frac{1}{n}} - 1$$

To confirm the anthelmintic efficacy results, necropsy worm counts were formally analysed using two-sided two sample *t*-tests for the pairwise comparison of each treatment group to the control group. The level of significance was set to *α* = 0.05.

As secondary preventive efficacy criteria the following parameters were evaluated statistically: (i) The individual maximum larvae counts between SD 114 and SD 130 in the treatment groups (G1-G3) were compared to the control group (G4) using a two-sided two-sample *t*-test with the level of significance set to *α* = 0.05. To evaluate the percentage preventive efficacy against faecal larvae excretion, GM and efficacy reduction of the maximum larvae counts in the study groups were calculated using the formula given above. (ii) The respiratory frequencies examined on the day prior to the first treatment (SD –1), the day prior to experimental infection (SD 83) and twice weekly between SD 85–128 were summarized and graphically displayed. Respiratory frequencies were also retrospectively compared pairwise between each treatment group (G1, G2 and G3) and the control group (G4) using *t*-tests with the level of significance set to *α* = 0.05 (two-sided). (iii) The lung pathology scores at necropsy (scores 0 to 4) of the treated groups (G1–G3) were compared to the control group (G4) using Wilcoxon’s rank sum test (two-sided) with the level of significance set to *α* = 0.05.

Other noted respiratory parameters (intensity and quality of respiratory sound, panting, abdominal involvement and coughing or retching) were evaluated but not analysed statistically.

## Results

### Inclusion criteria and safety assessment

All 31 cats met the criteria to be included in the study. After application of Bravecto® Plus spot-on solution for cats, one animal in G1 (ID 9657) exhibited slight scaling/flaking at the administration site for 1 day, and another two animals in G2 (ID 3599 and 6242) showed signs of itching (increased scratching and licking) at the administration site 2 h post treatment, which however disappeared the next day. No adverse effects on general health were observed in any of the cats.

### Faecal larvae counts

Beginning at 32–40 dpi, all cats of the control group (G4) excreted *A. abstrusus* L1 and remained constantly positive throughout the study. By contrast, only one animal of G1 excreted larvae at consecutive days, starting at 39 dpi until 46 dpi, when the last faecal examination was performed. Additionally, two further cats of G1, three of G2 and two of G3 were copromicroscopically positive on a single occasion between 36–44 dpi. In positive cats, the maximum number of excreted larvae in 5 g faeces on a single day varied between 54 and 45,900 L1 in the control group (G4), 1 and 31 (G1), 1 and 25 (G2), and 1 and 9 (G3) L1 in the treatment groups. Detailed data on individual cats are provided in Table 2.

**Table 2:** Parasitological parameters, lung pathology and preventive drug efficacy against larvae excretion and (pre-)adult of *Aelurostrongylus* *abstrusus*

Group	Animal ID	First day of L1 excretion (dpi)	Max. L1 count/day in 5 g faeces	GM of max. L1 count/day	Efficacy (%)	Lung pathology score	(Pre-)adult worm count	GM of adult worms	Preventive efficacy (%)
1 (treated 12 weeks prior to infection)	0801	n.a.	0	1.10	99.99	1	0	0.09	99.66
	1054	39	5			0	0		
	2936	n.a.	0			1	0		
	3881	37	1			0	0		
	5300	n.a.	0			1	1		
	6615	n.a.	0			0	0		
	8860	39	31			1	0		
	9657	n.a.	0			0	0		
2 (treated 8 weeks prior to infection)	0370	n.a.	0	1.19	99.99	0	0	0.00	100
	3599	36	1			0	0		
	5820	n.a.	0			2	0		
	6242	37	25			1	0		
	6554	n.a.	0			0	0		
	7273	42	9			0	0		
	7707	n.a.	0			1	0		
	8219	n.a.	0			0	0		
3 (treated 4 weeks prior to infection)	0862	n.a.	0	0.53	99.98	0	0	0.00	100
	1160	n.a.	0			0	0		
	1291	n.a.	0			0	0		
	3550	41	1			1	0		
	6179	44	9			0	0		
	6676	n.a.	0			0	0		
	9914	n.a.	0			1	0		
4 (untreated control)	1151	33	45,900	7574.29	99.99	3	23	26.57	n.a.
	1465	36	7400			3	36		
	1509	40	54			4	10		
	1588	38	1200			3	15		
	6407	33	43,100			4	64		
	6646	33	45,300			3	56		
	9013	36	11,300			3	23		
	9335	32	21,900			3	23		

dpi = day post infection, max. = maximum, GM = geometric mean, n.a. = not applicable

The GM of the maximum number of excreted larvae in 5 g faeces per day was 7574.29 (ranging from 54 to 45,900 L1) in the control group (G4) compared to 1.10 (G1), 1.19 (G2) and 0.53 (G3) in the treatment groups. The respective arithmetic mean (AM) was 22,019.25 in the control group (G4) and 4.63 (G1), 4.38 (G2) and 1.43 (G3) in the treatment groups. Based on GMs, the preventive efficacy of the treatment against *A. abstrusus* L1 excretion was 99.99% when applied 12 (G1) or 4 (G3) weeks and 99.98% when applied 8 (G2) weeks before experimental infection.

### Respiratory assessment and lung pathology

**Fig. 1:**
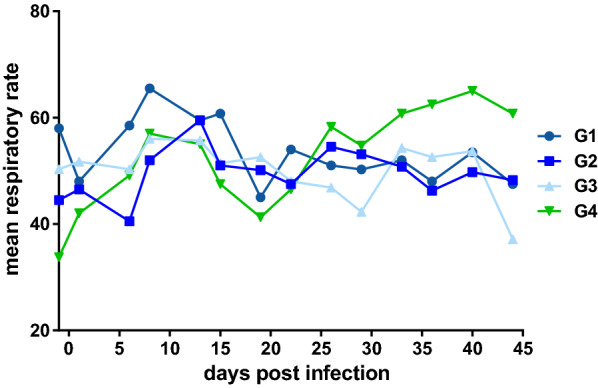
Mean respiratory frequency of the cats per study group as of the day (SD 83) prior to experimental infection with L3 of *Aelurostrongylus* *abstrusus*. G1: treatment 12 weeks before experimental infection, G2: treatment 8 weeks before experimental infection, G3: treatment 4 weeks before experimental infection, G4: control group

The mean respiratory frequency in the control group (G4) was higher than in the treatment groups (G1–G3, Fig. 1) starting at SD 110 (26 dpi). However, there was no statistically significant difference between the groups. The intensity and quality of inspiratory and expiratory sounds showed no obvious difference between the study groups throughout the course of the study. Panting was observed in three cats at day 13 pi, thereof each one in G1 (ID 9657), G2 (ID 6242) and G4 (ID 1465). In one animal of the control group (G4, ID 1465) coughing during the respiratory examination at day 26 pi was noticed. None of the study cats showed abdominal involvement.

**Fig. 2:**
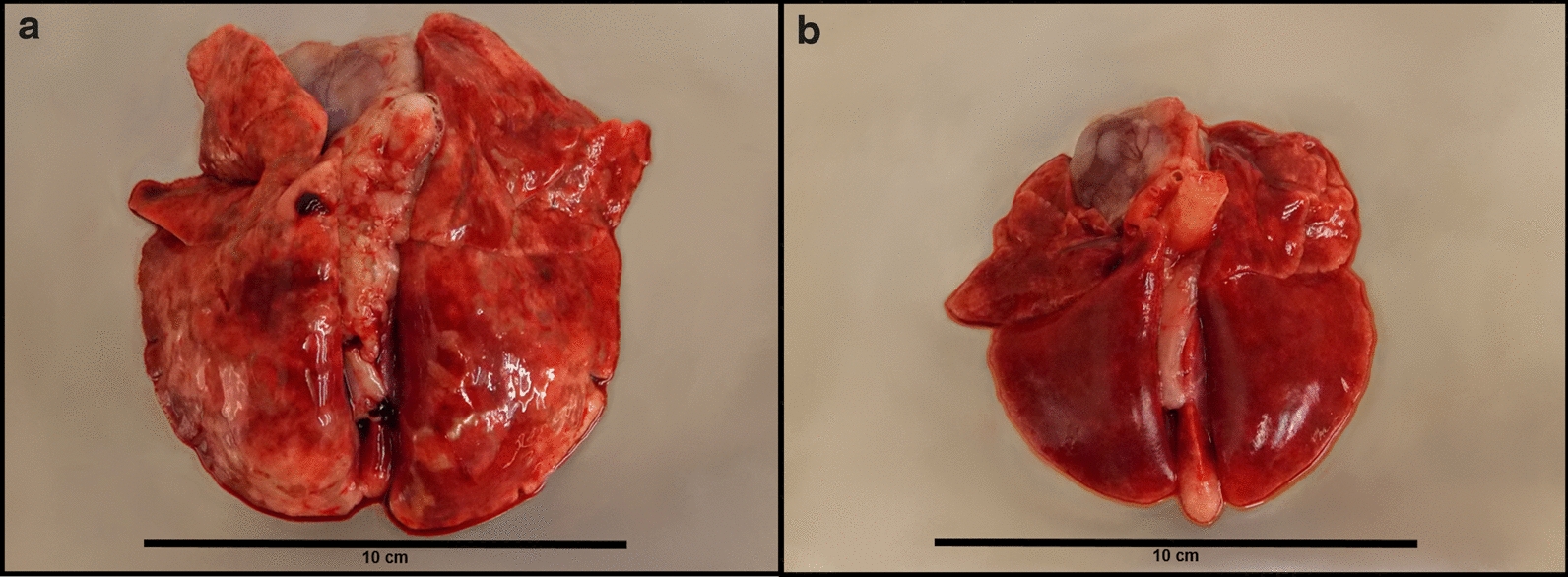
Lungs of cats experimentally challenged with 300 *Aelurostrongylus abstrusus* L3. **A** Lung example of the control group. Very severe verminous pneumonia with visible diffuse areas of hyperaemia, emphysema and consolidation, sub-pleural nodules and enlarged lymph nodes. **B** Lung example of Group 2 (treated 4 weeks before infection). No gross pathological signs of verminous pneumonia

**Fig. 3:**
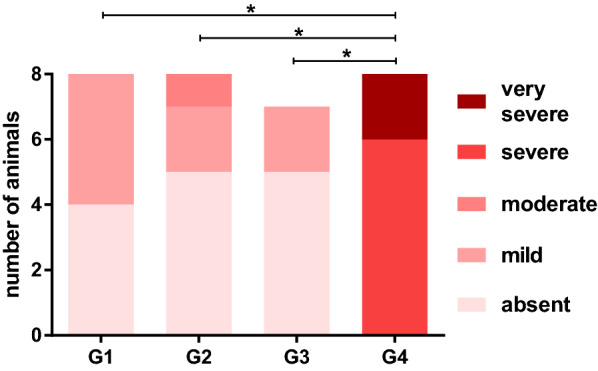
Lung pathology score in the four study groups at the day of necropsy (47-50 dpi, SD 131-134). G1: treatment 12 weeks before experimental infection, G2: treatment 8 weeks before experimental infection, G3: treatment 4 weeks before experimental infection, G4: control group. The asterisks indicate statistically significant differences in pathology scores between the control (G4) and the treatment (G1–G3) groups

The lungs of all eight control cats (G4) showed severe or very severe macroscopic signs of a verminous pneumonia, e.g. atelectasis, subpleural nodules and/or emphysema at necropsy (Fig. 2). By contrast, in 94.44% of the treated cats lung pathology was considered absent or mild. In G1, four lungs were rated as mildly altered, while no alterations were observed in the other four lungs. In G2, two lungs showed mild and one moderate pathological changes, whereas in the five remaining lungs pathological alterations were absent. In G3, two lungs were rated as mildly altered; the other five lungs appeared inconspicuous (Fig. 3, individual cat data are listed in Table 2). The mean lung pathology score in the control group was significantly higher than those of the treatment groups (*P* ≤ 0.0002).

### Nematode counts

According to the applied guidelines [34, 35], the experimental *A. abstrusus* infection was considered adequate, as all animals of the control group (G4) were infected and numbers of (pre-)adult worms retrieved from the lungs ranged from 10 to 64. In the treatment groups, one animal of G1 harboured one female worm, whereas no worms were detected in the cats belonging to G2 and G3. Individual cat data are provided in Table 2. The GM count was 26.57 worms in the control group (G4) compared to 0.09 (G1) and 0.00 (G2 and G3) worms in the treatment groups. The respective AM was 31.25 worms in the control group (G4) as well as 0.13 (G1) and 0.00 (G2 and G3) worms in the treatment groups. Based on GMs, the preventive efficacy of the treatment against *A. abstrusus* worms was 99.66% in G1 and 100% in G2 and G3, respectively. Statistical comparison showed significant differences (*P* < 0.0001) in necropsy worm counts between the control group and all treatment groups.

## Discussion

This is the first study demonstrating a long-term preventive efficacy of a drug against cat aelurostrongylosis when applied as a single treatment prior to infection. Based on necropsy worm counts, the preventive efficacy of Bravecto® Plus spot-on solution for cats (280 mg/ml fluralaner and 14 mg/ml moxidectin) at the minimum recommended dose of 2.0 mg moxidectin + 40 mg fluralaner/kg BW against aelurostrongylosis was 100% after application 8 or 4 weeks prior to experimental challenge infection and 99.66% when applied 12 weeks prior to infection. In the latter group, only one cat harboured a single adult *A. abstrusus* female with no faecal larvae excretion observed in this animal during the course of the study. However, another animal in this study group was positive for L1 excretion on several consecutive days. Thus, it appears certain that this cat had a patent infection, even though no adult lungworms could be retrieved from the lungs during necropsy. This is not surprising given that the small size (i.e. 5–10 mm) of adult worms [16, 39] and their location in the terminal bronchioles, alveoli and alveolar ducts (i.e. the lung parenchyma) [31, 39] render difficult to find *A. abstrusus* during manual dissection of the lungs [1, 16]. Therefore, it is not unlikely that single specimens were missed in the present study despite utmost care. This might also be true for the seven treated cats showing larvae excretion at singular days. Nonetheless, the maximum number of larvae in 5 g faeces ranged from 1 to 31 in the treated cats, while the control cats reached up to 45,900 L1. Overall, data evaluation resulted in > 99.9% preventive efficacy of Bravecto® Plus spot-on solution against larval excretion upon challenge infection 4–12 weeks after treatment.

A known issue of the experimental *A. abstrusus* infection model (inoculation of digested snail solution) is vomiting after inoculation [1, 33, 40]. Here, 23 cats vomited despite the administration of metoclopramide. One cat vomited within 1 h (46 min) after the infection and thus was re-inoculated. The other 22 cats vomited between 81 min pi and the next morning and were not re-inoculated, which might raise the question whether these animals kept the whole infection dose. Even though no precise data are available on how fast *A. abstrusus* L3 penetrate the intestinal mucosa, it can be assumed that the whole infection dose is assured as the mean half-time of gastric emptying after liquid meals is supposed to be < 80 min [41]. In addition, gastric emptying is accelerated by metoclopramide [42]. In another study, one cat vomited 23 min after inoculation of 800 L3, but still harboured 36 worms in the dissected half of the lung without re-inoculation [1]. This is in line with the findings in the present study, where seven out of eight cats of the control group vomited at some point after inoculation, but were all adequately infected with ten or more worms during necropsy without re-inoculation.

Notably, recurring outbreaks of a mild, apyretic upper respiratory tract disease (commonly known as “cat flu”) occurred in 13 cats (2 in G1, 4 in G2, 3 in G3, 4 in G4) during the whole course of the study. Upon request, the breeder reported similar events in the breeding facility. Possible underlying agents are feline herpesvirus, feline calicivirus, *Chlamydophila felis* or *Bordetella bronchiseptica* [43]. Affected cats, some of which were affected more than once, showed nasal and ocular discharge, sneezing, sniffling, stertor due to mucosal secretions and coughing. These signs could have interfered with the clinical signs because of experimental lungworm infection. Thus, the results of the respiratory assessment performed twice weekly after infection were probably overlain by the cat flu symptoms and could explain why there was no clear difference in respiratory parameters between the control and the treated groups. Furthermore, the respiratory assessments were not always carried out by the same veterinarian. In this respect, it should be taken into account that the evaluation of the intensity of respiratory sounds in cases of minor deviations from the physiological status cannot be fully objectified and thus not completely standardized between different examiners. In addition, subclinical or mild *A. abstrusus* infections are not unusual and may remain unnoticed by the owners or even veterinarians [2, 5, 6]. Hence, respiratory sounds and alterations should be considered with caution to assess the preventive efficacy of anthelmintics against *A. abstrusus*. Here, no statistically significant differences regarding the respiratory frequency were observed between the control and treatment groups, although the control animals showed an increased mean respiratory frequency, which may be interpreted as a first sign of increased oxygen demand or decreased gas exchange as in the case of aelurostrongylosis [44]. Further evaluations are warranted to assess whether the (mean) respiratory frequency or other clinical signs may be considered as auxiliary parameters in efficacy studies.

In contrast to the clinical parameters, statistically significant differences between the groups in gross lung pathology were observed, despite the presence of the mild upper respiratory tract infection in some cats. Since none of these cats showed fever or general depression, a pneumonia due to the “cat flu” was unlikely. Nevertheless, a minor impact on the lung pathology score cannot be excluded. However, the study groups were evenly affected by the “cat flu,” while lung appearance during necropsy showed clear differences: In the control group, the lungs showed severe to very severe pathological findings typical of aelurostrongylosis such as emphysema, subpleural opaque or yellowish nodules and areas of hyperaemia [1, 33, 40]. In all treated groups such findings were either absent or only mild to moderate. Thus, lung pathology appeared to be a suitable additional parameter to assess the preventive efficacy, and the observed data fit with a previous study, which reported marked differences in the gross lung appearance between control cats and those preventively treated against aelurostrongylosis [33]. This latter study showed the efficacy of two treatments of an imidacloprid 10%/moxidectin 1% spot-on formulation (Advocate®; Bayer Animal Health [part of ELANCO Animal Health]) at the minimum recommended dose of 10 mg/kg BW imidacloprid and 1 mg/kg BW moxidectin applied at a 4-week interval in preventing cat aelurostrongylosis [33]. The first treatment was performed either 4, 10 or 20 days before and the second 24, 14 or 4 days after experimental infection, respectively. As a single administration of moxidectin is reported to be effective at least in terms of suppressing larval excretion and resolving clinical signs in a patent infection [2, 6, 45], the protective effect of the first treatment in the mentioned study [33] applied prior to the challenge infection cannot be properly assessed. Therefore, it remains unclear whether and to what extent the treatment prior to infection had a preventive effect against subsequent challenge infection. Nevertheless, this study showed that monthly administration of the product reliably eliminated early larval stages and thereby precludes lung damages and patent infections by *A. abstrusus* [33].

The preventive efficacy of a single application of Bravecto® Plus spot-on solution for cats against aelurostrongylosis for a period of 12 weeks is in line with the treatment interval recommended by the manufacturer to control flea and tick infestations and to prevent heartworm disease. Similarly, at least four anthelmintic treatments or faecal examinations (and treatment in case of positive results) per year are recommended by the European Scientific Counsel Companion Animal Parasites (ESCCAP) to reduce the risk of excretion of *Toxocara* eggs in free-roaming cats [46]. However, for cats sharing homes with young children or immunocompromised persons, monthly treatment against roundworms or faecal examination is advised. A monthly interval is also generally recommended by the current country-specific German adaptation of the ESCCAP guidelines for cats with non-supervised outdoor access [47].

## Conclusion

The administration of Bravecto® Plus spot-on solution for cats (280 mg/ml fluralaner and 14 mg/ml moxidectin) 4, 8 or 12 weeks prior to *A. abstrusus* infection was safe and reliable in inhibiting the establishment of adult *A.* *abstrusus* in the lung and in preventing visible lung damages and larval excretion. Thus, it can be concluded that a single treatment prevents cat aelurostrongylosis for at least 12 weeks.

## Supplementary Information

**Additional file 1.** Detailed information on the study population and composition of the study groups.

## Data Availability

Most data analysed during this study are included in the article. The remaining data from this clinical study are proprietary and maintained by MSD Animal Health.

## Abbreviations

AM: Arithmetic mean
BW: Body weight
dpi: Days post infection
ESCCAP: European Scientific Counsel for Companion Animal Parasites
GM: Geometric mean
G: Study group
L1: First-stage larvae
L3: Third-stage larvae
L4: Fourth-stage larvae
L5: Fifth-stage larvae
pi: Post infection
SD: Study day
VICH: International Cooperation on Harmonization of Technical Requirements for Registration of Veterinary Medicinal Products
WAAVP: World Association for the Advancement of Veterinary Parasitology
