# Supplementary material for: Efficacy of Bravecto® Plus spot-on solution for cats (280 mg/ml fluralaner and 14 mg/ml moxidectin) for the prevention of aelurostrongylosis in experimentally infected cats
Source: Parasit Vectors. 2021 Feb 16;14:110. doi: 10.1186/s13071-021-04610-y (PMC7885211; doi:10.1186/s13071-021-04610-y)
Supplement: Supplementary file 1 — Additional file 1. Detailed information on the study population and composition of the study groups. [file 13071_2021_4610_MOESM1_ESM.docx]

**Supplementary Table 1:** Detailed information on the individual cats and composition of the study groups at inclusion in the study (study day 0).

| **Group** | **Animal ID** | **Sex** | **Age at SD 0 (weeks)** | **Mean age at SD 0 (weeks)** | **Bodyweight at SD -1 (kg)** | **Mean bodyweight at SD -1 (kg)** |
| --- | --- | --- | --- | --- | --- | --- |
| 1  (treated 12 weeks prior to infection) | 0801 | male | 29 | 26 | 4.2 | 3.0 |
|  | 1054 | female | 26 |  | 2.4 |  |
|  | 2936 | male | 23 |  | 2.9 |  |
|  | 3881 | female | 29 |  | 2.5 |  |
|  | 5300 | female | 27 |  | 2.6 |  |
|  | 6615 | male | 26 |  | 3.1 |  |
|  | 8860 | male | 27 |  | 3.1 |  |
|  | 9657 | male | 22 |  | 2.9 |  |
| 2  (treated 8 weeks prior to infection) | 0370 | male | 29 | 25 | 4.1 | 2.9 |
|  | 3599 | female | 23 |  | 2.8 |  |
|  | 5820 | female | 24 |  | 2.5 |  |
|  | 6242 | female | 24 |  | 2.9 |  |
|  | 6554 | male | 26 |  | 3.0 |  |
|  | 7273 | male | 21 |  | 2.9 |  |
|  | 7707 | female | 28 |  | 2.3 |  |
|  | 8219 | female | 23 |  | 2.3 |  |
| 3  (treated 4 weeks prior to infection) | 0862 | male | 29 | 26 | 3.4 | 2.9 |
|  | 1160 | female | 26 |  | 2.2 |  |
|  | 1291 | female | 26 |  | 2.3 |  |
|  | 3550 | male | 21 |  | 2.9 |  |
|  | 6179 | male | 27 |  | 3.6 |  |
|  | 6676 | male | 26 |  | 3.1 |  |
|  | 9914 | female | 29 |  | 2.8 |  |
| 4  (untreated control) | 1151 | female | 27 | 26 | 2.4 | 2.9 |
|  | 1465 | male | 24 |  | 3.1 |  |
|  | 1509 | male | 26 |  | 3.9 |  |
|  | 1588 | female | 26 |  | 2.7 |  |
|  | 6407 | female | 27 |  | 3.0 |  |
|  | 6646 | female | 27 |  | 2.4 |  |
|  | 9013 | female | 24 |  | 2.4 |  |
|  | 9335 | male | 21 |  | 3.1 |  |

SD = study day
